# Recruitment of Rpd3 to the Telomere Depends on the Protein Arginine Methyltransferase Hmt1

**DOI:** 10.1371/journal.pone.0044656

**Published:** 2012-08-31

**Authors:** Eric J. Milliman, Neelu Yadav, Yin-Chu Chen, Bhavana Muddukrishna, Sheelarani Karunanithi, Michael C. Yu

**Affiliations:** Department of Biological Sciences, State University of New York at Buffalo, Buffalo, New York, United States of America; Tulane University Health Sciences Center, United States of America

## Abstract

In the yeast *Saccharomyces cerevisiae*, the establishment and maintenance of silent chromatin at the telomere requires a delicate balance between opposing activities of histone modifying enzymes. Previously, we demonstrated that the protein arginine methyltransferase Hmt1 plays a role in the formation of yeast silent chromatin. To better understand the nature of the Hmt1 interactions that contribute to this phenomenon, we carried out a systematic reverse genetic screen using a null allele of *HMT1* and the synthetic genetic array (SGA) methodology. This screen revealed interactions between *HMT1* and genes encoding components of the histone deacetylase complex Rpd3L (large). A double mutant carrying both *RPD3* and *HMT1* deletions display increased telomeric silencing and Sir2 occupancy at the telomeric boundary regions, when comparing to a single mutant carrying Hmt1-deletion only. However, the dual *rpd3/hmt1*-null mutant behaves like the *rpd3*-null single mutant with respect to silencing behavior, indicating that *RPD3* is epistatic to *HMT1*. Mutants lacking either Hmt1 or its catalytic activity display an increase in the recruitment of histone deacetylase Rpd3 to the telomeric boundary regions. Moreover, in such loss-of-function mutants the levels of acetylated H4K5, which is a substrate of Rpd3, are altered at the telomeric boundary regions. In contrast, the level of acetylated H4K16, a target of the histone deacetylase Sir2, was increased in these regions. Interestingly, mutants lacking either Rpd3 or Sir2 display various levels of reduction in dimethylated H4R3 at these telomeric boundary regions. Together, these data provide insight into the mechanism whereby Hmt1 promotes the proper establishment and maintenance of silent chromatin at the telomeres.

## Introduction

In a eukaryotic cell, selective transcriptional repression or silencing dictates the accessibility of specific chromatin domains by the transcriptional machinery; this results in varying degrees of transcriptional competency across the eukaryotic genome. Euchromatin refers to the chromatin regions at which transcription is generally active, whereas heterochromatin refers to those that are largely devoid of transcriptional activity (reviewed in [1]). In the budding yeast *S. cerevisiae,* three chromosomal regions are epigenetically silenced with respect to transcription: the telomeres, the silent mating loci (*HMR* and *HML*), and the ribosomal DNA (rDNA) repeats (reviewed in [2], [3]). Critical to the establishment and maintenance of these heterochromatic regions are *cis*-acting elements such as silencers and the telomeric repeats, both of which serve as nucleation points to recruit *trans*-acting proteins. *Trans*-acting proteins, such as those of epigenetic modification machinery, both initiate and regulate the spreading of repressive chromatin (reviewed in [4], [5], [6]). It is thought that the delicate balance of opposing enzymatic activities of chromatin-opening and chromatin-condensing complexes determines the position of the boundary between the euchromatin and heterochromatin. This mechanism is exemplified by the *S. cerevisiae* telomeric heterochromatin, in which a flexible boundary is established by the chromatin-opening activities of the histone acetyltransferase (HAT) complex SAS-I and the chromatin-condensing activities of NAD^+^-dependent histone deacetylase (KDAC) Sir2 (silent information regulator-2) [7], [8].

Rpd3 is a member of the class I KDACs in *S. cerevisiae*, and has been shown to deacetylate lysines on both histone H3 and H4 [9]. Through its lysine-modifying activities, Rpd3 represses transcription at many of the individual loci that have been examined. For example, Rpd3 deacetylates histones at the promoters of *INO1* and *IME2* to repress their transcription [10], [11]. However, Rpd3 can also promote the transcription of specific genes, such as *MSB2*, by binding to their promoters [12]. Loss of Rpd3 increases silencing at all three silent chromatin regions, i.e. the telomeres [13], [14], rDNA repeats [9], [14], and silent mating loci [15]. In a recent study, it was proposed that Rpd3 antagonizes Sir2-dependent propagation of silent chromatin in wild-type cells, and that its loss results in defective chromatin boundary formation because this allows Sir-dependent local propagation of the silent state [16]. Mechanistically, this may be achieved by removing Sir2 substrates [17]. Nevertheless, Rpd3 is able to cooperate with Esa1, an enzyme whose enzymatic activity is opposite that of Sir2, to regulate transcription by promoting the acetylation of lysine 12 of histone H4 (H4K12) [18].

Arginine methylation is a post-translational modification commonly found in nucleic acid-binding proteins, and is catalyzed by a family of enzymes called protein arginine methyltransferases (PRMTs) (reviewed in [19], [20]). Arginine residues can be either monomethylated or dimethylated by specific types of PRMT: type I PRMTs catalyze the formation of asymmetric dimethylarginines (ADMAs), whereas type II PRMTs catalyze the formation of symmetric dimethylarginines (SDMAs). As a consequence of this post-translational modification, the binding affinity of methylated protein is often altered for either another protein or a nucleic acid target [21]. For example, loss of arginine methylation alters the biochemical association between components of the messenger ribonucleoprotein particle (mRNP) during its biogenesis [22], [23]. In addition, the methylation status of the Src kinase substrate Sam68 is required for its ability to interact with SH3-containing proteins [24]. In terms of nucleic acid targets, the mRNA export factor REF/ALY exhibits reduced RNA-binding capacity in the methylated state, thereby ensuring that the message is handed off to the mRNA export factor TAP [25]. Arginine methylation also occurs on histone tails [26], [27], [28], [29], [30], [31], and this modification likely generates a site that allows docking of effector proteins. As these proteins subsequently help recruit additional components of the epigenetic modification machinery, this methylation step can contribute to the activation or repression of gene transcription.

Hmt1 (also termed Rmt1) has been identified as the only type I PRMT in *S. cerevisiae*, and is the functional homolog of mammalian PRMT1 [32], [33]. In mammalian cells, PRMT1 specifically dimethylates histone H4 at arginine 3 (H4R3), and this modification is associated with the transcriptional activation of a number of loci [30], [31]. Hmt1 can dimethylate both free histone H4R3 from yeast and histone H2A at arginine 3 from human *in vitro*, yet its loss does not appear to affect bulk methylation of H4R3 in yeast *in vivo* [34]. Mutants lacking either Hmt1 or its catalytic activity display a loss of telomeric silencing, as well as decreases in Sir2 occupancy and H4R3 dimethylation at both telomeric boundary regions and silent mating loci [35]. These mutants also display an increase in the level of histone H4 acetylation within these regions, although the specific residue(s) contributing to this change is unknown [35]. It is possible that the specific chromosomal context in which H4R3 methylation occurs may provide the cue needed for recruitment of the epigenetic modification machinery during either the activation or repression of gene expression. Understanding the nature of the interplay between Hmt1 and other components of the epigenetic modification machinery is expected to provide important insight into the role of Hmt1 in the formation and maintenance of yeast silent chromatin.

In the current study, we used a null allele of *HMT1* in conjunction with the synthetic genetic array (SGA) methodology to systematically and comprehensively screen for all non-essential yeast genes that interact with *HMT1*, and generated an *HMT1* genetic interaction network based on our findings. Gene ontology (GO) analysis of our SGA data showed that *HMT1* interacts with genes encoding various components of the Rpd3L (large) complex. In the Hmt1 loss-of-function mutants, recruitment of Rpd3 to the telomeric boundary region is increased. In mutants carrying both Rpd3 and Hmt1 deletions, increased silencing at the telomere and increased Sir2 recruitment at telomeric boundary region is observed compared to Δ*hmt1* mutants. The Hmt1 loss-of-function mutants display a decrease in the levels of H4K5 acetylation (a known Rpd3 substrate) and an increase in the levels of acetylated H4K16 (a known Sir2 substrate) at the telomeric boundary regions. Finally, mutants lacking either Sir2 or Rpd3 display a decrease in the levels of dimethylated H4R3 at the telomeric boundary regions, albeit to a different degree. Overall, our results indicate that Hmt1 has the potential to influence the recruitment and actions of KDACs in order to promote the maintenance of silent chromatin.

## Results

### Systematic Genome-wide Reverse Genetic Screen of *HMT1* Using Synthetic Genetic Array Analysis

To obtain further insight into the interactions between *HMT1* and other genes, we conducted a synthetic genetic array (SGA) analysis using the Δ*hmt1* mutant as the query strain. This approach allowed us to construct and analyze double mutants in which the Δ*hmt1* mutation was combined with deletions in most of the non-essential genes of *S. cerevisiae*. Such information was expected to enable us to uncover any role Hmt1 may play within a broad biological network. The sensitivity of our screen was maximized by constructing the Δ*hmt1* query strain from a parental strain (15578-1.2b) developed by the Hartman lab [36]. This strain has been re-engineered from the original Boone lab SGA query strain, with the purpose of reducing causes of false negatives commonly found in SGA-type analyses; for example, mating-type-regulated escape from auxotrophy and mating-type switching. Thus, the use of this query strain in our SGA analysis was expected to result in a reduction in the overall number of false negatives, and thus a higher sensitivity.

**Figure 1:**
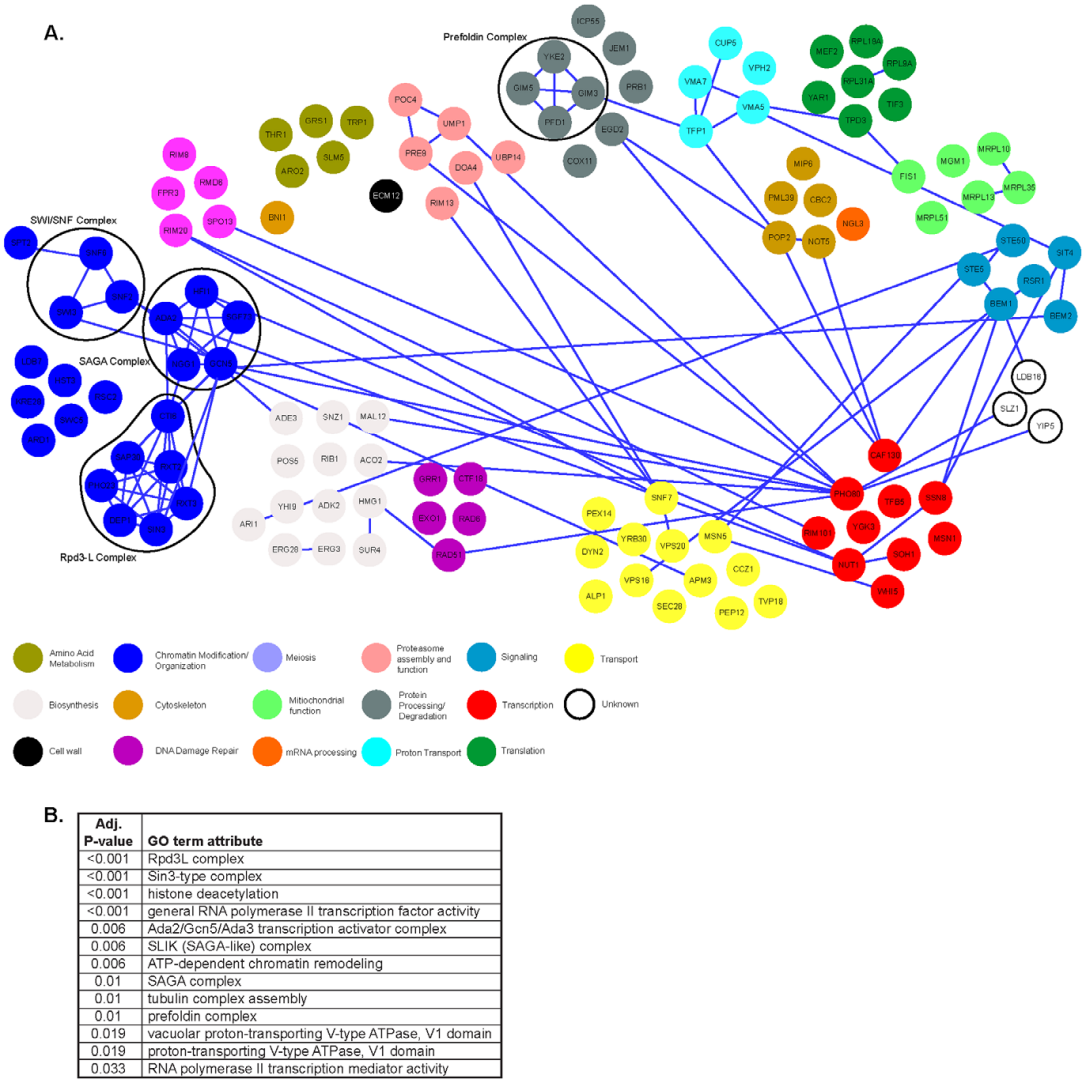
Synthetic Genetic Array Analysis of *HMT1*. **A)** HMT1 *genetic interaction network.* Physical interactions between *HMT1* and all of its genetic interactors identified from this study using the Synthetic Genetic Array methodology. The product of the gene identified is indicated within the relevant circle. The blue line represents a previously identified physical interaction. The complete genetic interaction network was created using Cytoscape, and the physical interaction data were obtained from the *Saccharomyces* Genome Database [55], [56]. **B)** *GO terms for the* Δ*hmt1 SGA interaction data set reveals enrichment for components of the Rpd3(L) complex* [57]. Overrepresented GO terms and corresponding adjusted p-values were determined using FuncAssociate 2.0. GO term attributes are listed based on ascending adjusted *p*-values [40], [58].

Our screen was carried out in triplicate, against an ordered array of approximately 4700 viable gene deletion strains. The relative growth of each double mutant was measured and analyzed using a MATLAB script slightly modified from that published by the Weissman lab [37]. Our SGA screen identified a total of 123 deletions that exhibited genetic interactions with Δ*hmt1* (Table S1 and Table S2). This number represents a two-fold increase over the interactions identified in previous SGA screens using the Boone lab query strain background [38], [39]. We only identified four genes in our screen had also been obtained in the previous screen: *GCN5*, *ARD1, ADA2,* and *CBC2* [38]. Of the 123 total genetic interactions we uncovered, 34 were growth enhancing, and 89 were growth suppressing. To determine the relationship between genes in the *HMT1* genetic interaction network, we queried each identified candidate against the *Saccharomyces* Genome Database (SGD) and compiled the known physical interactions into a list. These physical interactions were then superimposed on the *HMT1* genetic interaction dataset, and the overlap was graphically displayed (Fig. 1). This analysis suggested that Hmt1 participates in various biological processes whose effectors display a high level of interconnectivity.

### Multiple Subunits of Rpd3L Complex Display Genetic Interactions with *HMT1*

**Figure 2:**
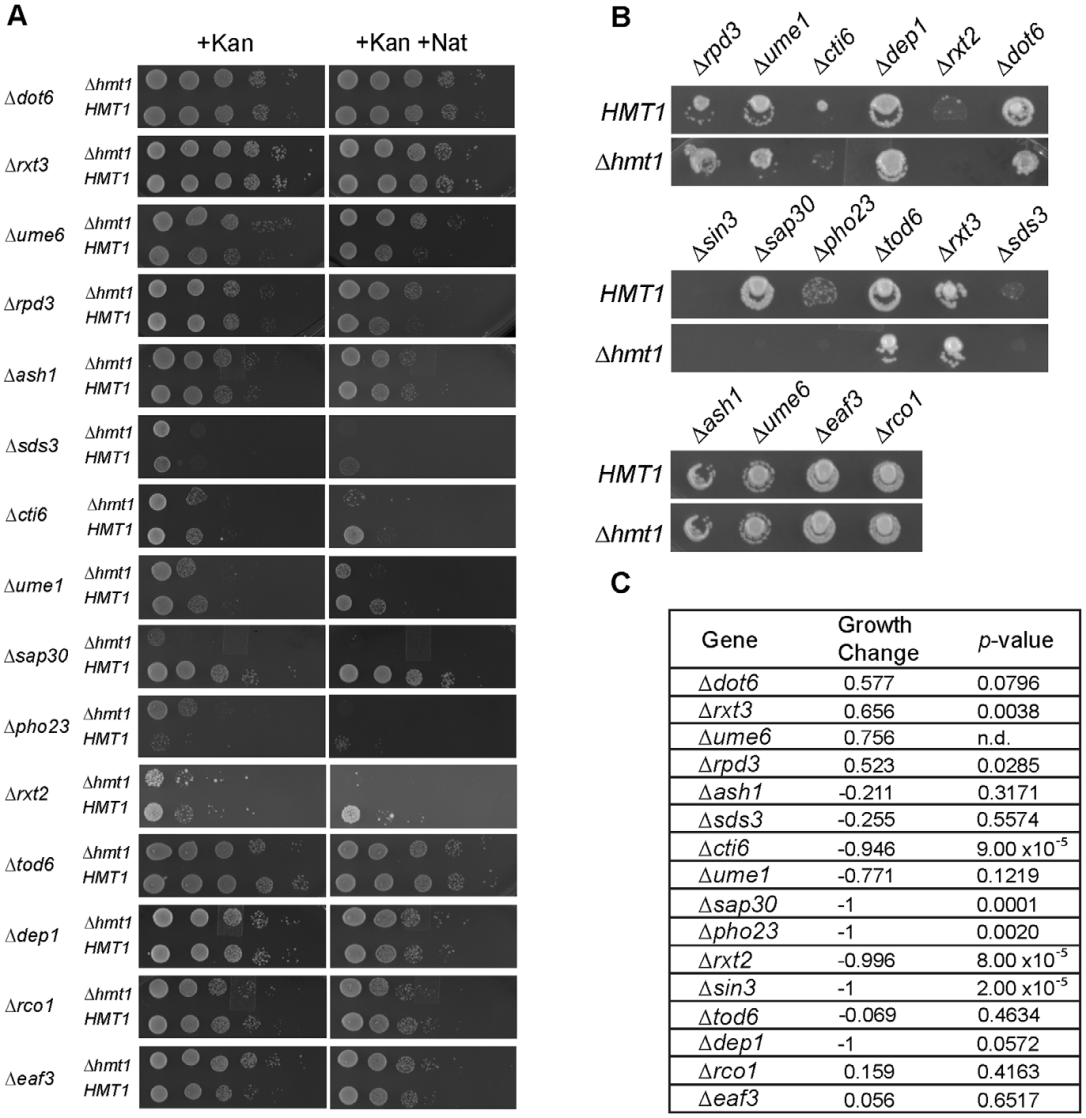
Genetic interactions between *HMT1* and genes encoding Rpd3L complex components. **A)** Spot assay of 10-fold serially diluted haploid cells with single Rpd3 complex component deletion (+Kan) or double mutant selection (+Kan+Nat) resulting from mating with either *HMT1* or Δ*hmt1* query strains. **B)** Growth of haploid, double-drug resistant strains produced from matings between strains deleted for genes encoding components of the Rpd3L complex, or components specific for Rpd3S complex (Δ*eaf3* and Δ*rco1*), with either *HMT1* or Δ*hmt1* query strains. **C)** Tabulated results of growth differences for Rpd3 complex components from genome-wide SGA screen.

Using software that identifies the enrichment of specific gene function groups based on annotated gene ontology (GO) terms [40], we found the attribute ‘Rpd3L complex’ (with adjusted *P-*value of less than 0.001) as one of the most enriched among the 123 genetic interactions identified in our SGA screen (Fig. 1B). Both positive (growth enhancing) and negative (lethal or growth inhibitory) genetic interactions were discovered for genes encoding the Rpd3L complex. We confirmed these results by carrying out an additional independent set of SGA experiments using just mutants of the Rpd3L complex and Rpd3S complex both by traditional SGA methodology (Fig. 2B) and by spotting assays (Fig. 2A). Of the 14 genes annotated in the *Saccharomyces* Genome Database (SGD) as ones encoding components of the Rpd3L complex, six displayed negative synthetic genetic interactions (Δ*sds3,* Δ*cti6,* Δ*ume1,* Δ*sap30,* Δ*pho23,* and Δ*rxt2*) with Δ*hmt1*, two displayed positive synthetic genetic interaction (Δ*rpd3* and Δ*ume6*) with Δ*hmt1*, and the other seven genes fell outside our significance criteria (Figs. 2A and 2B). In our original SGA screen, Δ*rxt3* showed a positive interaction while Δ*dep1* showed a negative interaction. However, neither showed an interaction upon confirmation (Figs. 2A and 2B, compare Δ*hmt1* to *HMT1* spots for Δ*rxt3* and Δ*dep1*). For Δ*sds3*, and Δ*ume6*, our follow-up screens indicated that they were false negatives (Figs. 2A and 2B, compare Δ*hmt1* to *HMT1* spots for Δ*sds3* and Δ*ume6*) as they had the same interaction type in the original SGA screen, but their *p-*values from the original SGA screen did not pass the cutoff criteria (Fig. 2C). Two negative controls used in the follow-up SGA experiment, Δ*eaf3* and Δ*rco1*, are components exclusive to the Rpd3S (small) complex, and they did not display any genetic interactions with Δ*hmt1* (Figs. 2A and 2B, compare *HMT1* and Δ*hmt1* rows for Δ*eaf3* and Δ*rco1*). Although Δ*sin3* resulted in a synthetic genetic interaction with Δ*hmt1* in the initial screen, this interaction could not be confirmed by our subsequent SGA analysis (Fig. 2B). Nevertheless, the clustering of negative and positive genetic interactions between genes that encode components of the Rpd3L complex and Δ*hmt1* suggests that Hmt1 has an influence on the activity of the Rpd3L complex.

### The Effects of Hmt1 on Rpd3 Occupancy Across the Telomeric Boundary Regions

Our SGA screen revealed interactions between genes encoding components of the Rpd3L complex and *HMT1*. The catalytic subunit of the Rpd3L complex, Rpd3, has a well-known role in the maintenance of silent chromatin at the *S. cerevisiae* telomere; mutants lacking *RPD3* display enhanced silencing in this region [9], [13]. Given that we previously demonstrated a role for Hmt1 in establishing and maintaining yeast silent chromatin at the telomere [35], we wanted to further investigate a potential role for an interaction between Hmt1 and the Rpd3L complex in this function.

Previously, a complex consisting of Rpd3 and Sin3 was shown to be specifically recruited to create a repressive chromatin domain *in vivo* [41]. Additionally, Rpd3 is recruited to both telomeres and silent mating loci [16]. Given that Hmt1 loss-of-function mutants display decreased Sir2 recruitment to the telomeric boundary region [35] and Rpd3 restricts the amount of Sir2 recruited to the same region [17], we wanted to determine whether Hmt1 function influences the level of Rpd3 recruited to the same telomeric boundary region.

**Figure 3:**
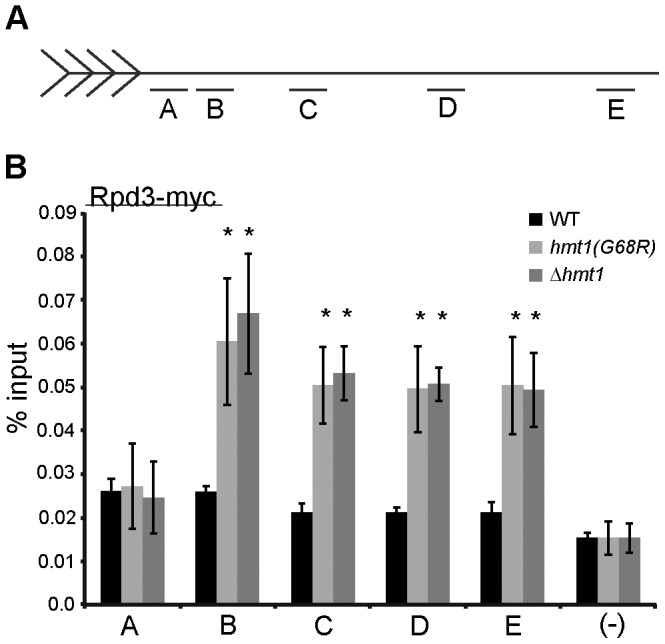
Rpd3 occupancy at telomeric boundary regions is increased in the Hmt1 loss-of-function mutants. **A)** Schematic representation of telomere VI-R and the quantitative PCR primer sets (A–E) for loci examined by ChIP in this study. **B)** *Rpd3 occupancy across the telomeric boundary region in Hmt1 loss-of-function mutants.* ChIP was performed using anti-Myc antibody to immunoprecipitate myc-tagged Rpd3 from wild-type, Δ*hmt1,* and *hmt1(G68R)* cells. Bars represent the experimental signal normalized to signal from a non-transcribed intergenic region (“(–) Ctrl”). Error bars represent standard deviation of three biological samples (n = 3) per genotype, and asterisks denote *p-*value of 0.05 by Student’s t-Test.

To test the effects of Hmt1 on the recruitment of Rpd3 to the telomeric boundary regions, we generated yeast strains that express a functional Rpd3 tagged with thirteen copies of C-terminal Myc epitope, in wild-type cells and various *hmt1* mutant backgrounds. Directed chromatin immunoprecipitation (ChIP) experiments were then performed to determine the effects Hmt1 has on the occupancy levels of Rpd3 at distances of 0.35, 0.6, 1.4, 2.8, and 5 Kb from the telomeric repeats (Fig. 3A). At a distance of 0.35 Kb from the telomeric repeats, no change in Rpd3 occupancy was observed, regardless of genetic background (Fig. 3B, compare black bar to light and dark gray bars in A). However, at a distance of 0.6 to 5 Kb from the telomeric repeats, Rpd3 occupancy was drastically increased in both of the Hmt1 loss-of-function mutants (Fig. 3B, compare black bars to light and dark gray bars in B to E). Based on the data from this ChIP assay, we conclude that Rpd3 recruitment to the telomeric boundary region can be influenced by the enzymatic activity of Hmt1 within a cell.

### The Epistatic Effects of Hmt1 and Rpd3 on Silencing and Sir2 Recruitment

**Figure 4:**
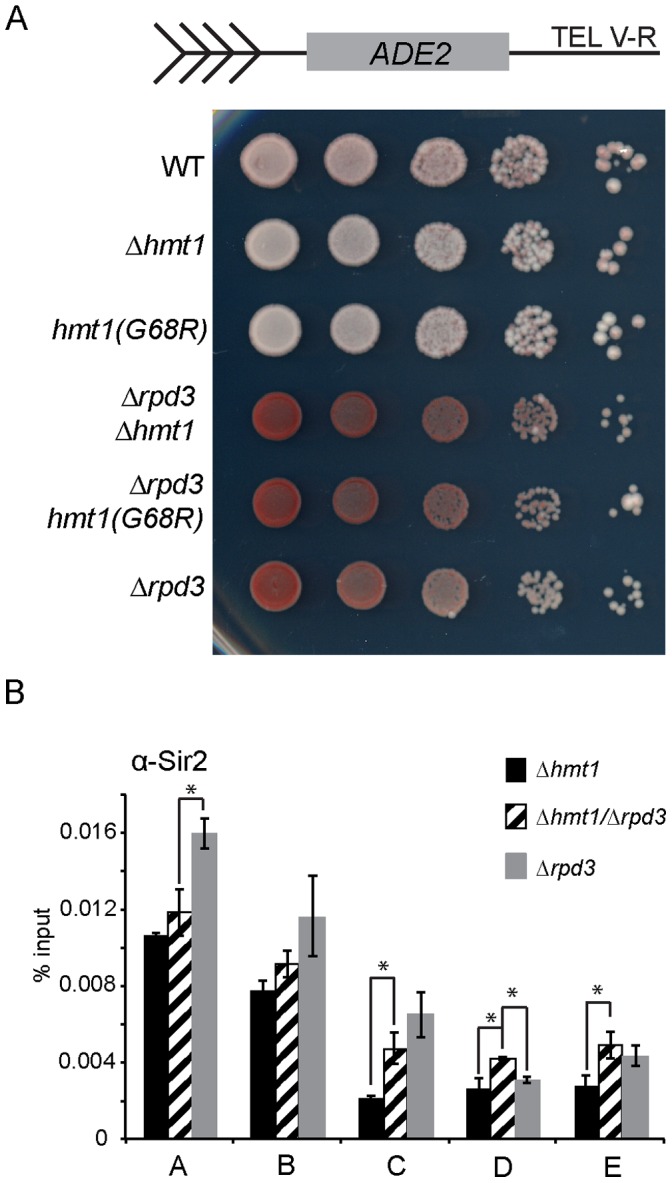
Epistatic analysis of silencing in Hmt1 and Rpd3 mutants. **A)** Telomeric Silencing Assay comparing Δhmt1/Δrpd3 to either Δhmt1 or Δrpd3 single mutant. **B)** Sir2 occupancy across the telomeric boundary region in Δhmt1, Δrpd3, or Δhmt1/Δrpd3 mutants. ChIP was performed using anti-Sir2 antibody to immunoprecipitate Sir2 from Δhmt1, Δrpd3, and Δhmt1/Δrpd3 cells. Primer sets are the same as in Fig. 3. Bars represent the experimental signal normalized to the GAL1 ORF. Error bars represent standard deviation of three biological samples (n = 3) per genotype, and asterisks denote p-value of 0.05 by Student’s t-Test.

Since Hmt1 and Rpd3 affect the dynamics of silent chromatin formation in opposing manners [17], [35], we decided to determine whether loss of Hmt1 could restore wild-type silencing in a Δ*rpd3* background. Using a established telomere silencing assay [42], we observe an increased telomeric silencing in Δ*rpd3* strain (Fig. 4A, compare WT to Δ*rpd3)* and loss of silencing in the Hmt1 loss-of-function strains (Fig. 4A, compare WT to Δ*hmt1* and *hmt1(G68R)*) as previously reported [14], [35], [43]. In the double mutants, however, these cells show increased silencing when compared to the wild-type, much like the Δ*rpd3* strain (Fig. 4A, compare WT to Δ*hmt1/*Δ*rpd3* and Δ*rpd3/hmt1(G68R)*. Thus, our results indicate that the Δ*rpd3* phenotype is dominant to the silencing defect caused by mutations in Hmt1.

To examine this relationship further at the molecular level, we used directed ChIP to compare the level of Sir2 occupancy in Δ*hmt1*, Δ*rpd3*, and Δ*hmt1*/Δ*rpd3* double mutant (Fig. 4B). We observed that Sir2 occupancy across the telomere proximal region is increased in the Δ*hmt1*/Δ*rpd3* double mutant when compared to the Δ*hmt1* mutant (Fig. 4B, compare Δ*hmt1* (black bars) to Δ*hmt1*/Δ*rpd3* (striped bars)). As previously seen, Sir2 recruitment increased minimally within 1 Kb of the telomeric end [16] (Fig. 4B, compare Δ*hmt1* (black bars) to Δ*hmt1*/Δ*rpd3* (striped bars), and Δ*hmt1* to Δ*rpd3* (gray bars), regions A and B) and this trend continues in both the Δ*hmt1*/Δ*rpd3* double mutant even at a distance greater than 1 Kb from the telomeric end (Fig. 4B, compare Δ*hmt1* (black bars) to Δ*hmt1*/Δ*rpd3* (striped bars) and Δ*hmt1* to Δ*rpd3* (gray bars), regions C, D, and E). Thus, our ChIP data support the results from the silencing assay in which we observed more silencing in the Δ*hmt1*/Δ*rpd3* double mutant strains when compared to the single Hmt1 mutant strain.

### Changes of Acetyl-H4K5 and Acetyl-H4K16 Levels at Telomeric Boundary Regions are Observed in *hmt1* Mutants

**Figure 5:**
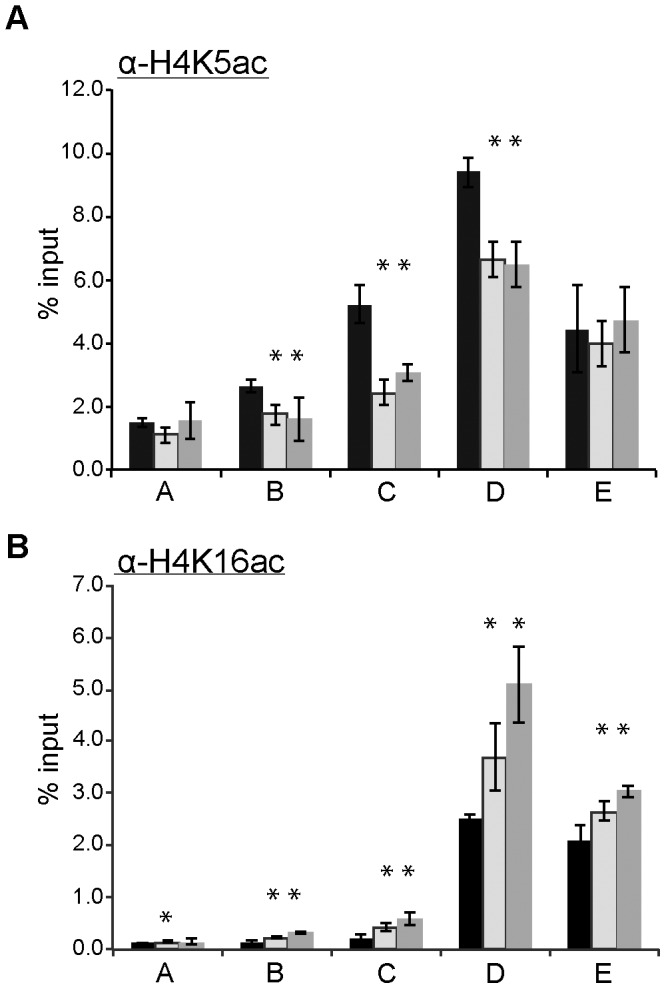
Hmt1 loss-of-function mutants display various changes in the occupancy of acetyl-K5 and -K16 of histone H4 at telomeric boundary regions. Directed ChIPs using anti-acetyl-H4K5 antibody **(part A)**, or anti-acetyl-H4K16 antibody **(part B)** in wild-type, Δ*hmt1,* and *hmt1(G68R)* cells. Primer sets used for this analysis were the same as those used in Fig. 3. Bars represent the experimental signals normalized to signal for the highly transcribed control, *ACT1.* Error bars represent standard deviation of three biological samples (n = 3) per genotype, and asterisks denote *p-*value of 0.05 by Student’s t-Test.

Since *rpd3-*null mutants display enhanced acetylation of H4K5 [44], we examined the level of acetylated H4K5 at the same telomeric boundary region in the Hmt1 loss-of-function mutants, using a ChIP-grade antibody that is specific for acetyl-H4K5 [45]. We found that the level of acetyl-H4K5 was decreased at a distance of 0.6 to 2.8 Kb away from the telomeric repeat (Fig. 5A, compare black bars to light and dark gray bars in B to D). However, at 5.0 Kb from the telomeric repeats, the level of acetylated H4K5 observed was unchanged in both of the *hmt1* mutants (Fig. 5A, compare black bars to dark gray bars or light gray bars in E). These data indicate that *hmt1* mutants are impaired in their ability to acetylate H4K5 across the telomeric boundary regions to 3 Kb from the chromosome end, when compared to the wild-type cells. Thus, our ChIP data indicate that Hmt1 functionality is important for maintaining wild-type levels of acetylation of H4K5 at the telomeric boundary region.

Given that the recruitment of Sir2 to the telomeric boundary region is also altered in the Hmt1 loss-of-function mutants [35], we wanted to examine how the Hmt1 functionality influences the levels of acetylated H4K16, which is a well-established substrate for Sir2 [46] in this region. To this end, we performed a ChIP assay (using a ChIP-grade antibody that is specific for acetyl-H4K16 [45]) in the Hmt1 loss-of-function mutants (Fig. 5B). Our data show that in both Hmt1 loss-of-function mutants, the level of acetyl-H4K16 was increased across all of the tested regions of the telomeric boundary (Fig. 5B, compare black bars to light and dark gray bars in A to E). The most pronounced increase in acetyl-H4K16 levels was observed at a distance of 2.8 Kb from the telomeric repeats (Fig. 5B, compare light and dark gray bars to black bars in D). These ChIP data support our previous observation with regard to a decrease in Sir2 occupancy in these Hmt1 loss-of-function mutants [35]. Furthermore, acetyl-H4K16 is likely one of the residues that contributes to the overall increase in the H4 acetylation previously observed in this region [35].

### Rpd3 and Sir2 are Required for Proper Levels of H4R3 Dimethylation at the Telomeric Boundary Region

**Figure 6:**
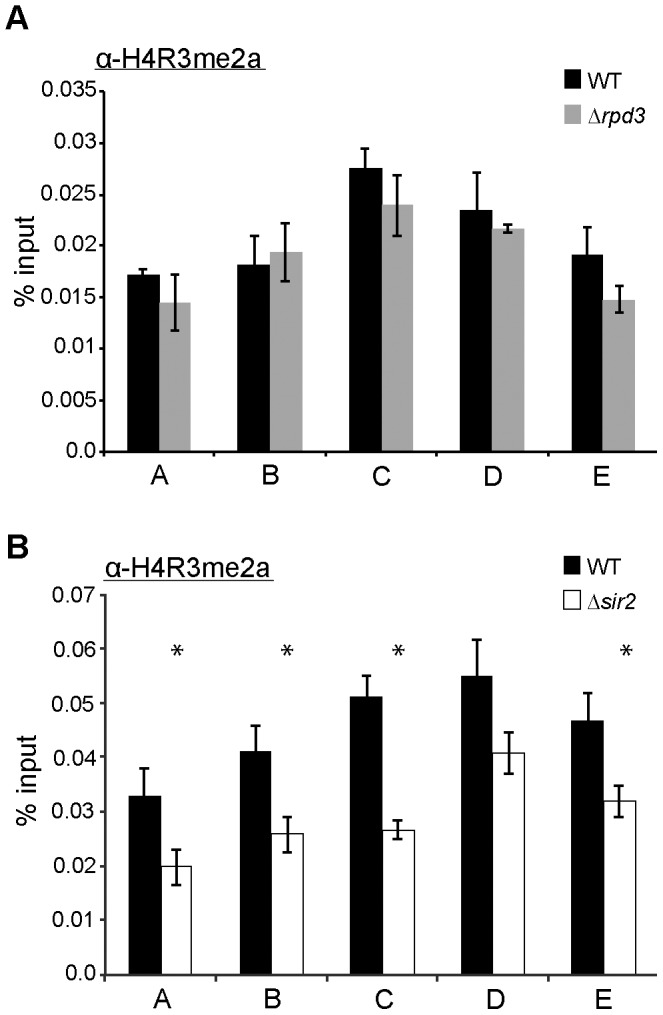
The effects of Rpd3 and Sir2 on H4R3 methylation. Directed ChIP was performed using anti-dimethyl-H4R3 (H4R3me2) antibody in wild-type, Δ*rpd3* **(part A)**, and Δ*sir2* **(part B)** cells. Primer sets used for this analysis were the same as in Fig. 3. Bars represent the experimental signals normalized to the highly transcribed control, *ACT1.* Error bars represent standard deviation of three biological samples (n = 3) per genotype, and asterisks denote *p-*value of 0.05 by Student’s t-Test.

*In vivo*, PRMT1-catalyzed H4R3 methylation is required for histone acetylation in mammalian cells, implying that H4R3 methylation cooperates with other histone modifications in regulating chromatin states [47]. In the absence of Hmt1, we have observed changes in the levels of Rpd3 (Fig. 3B) and Sir2 [35] (Fig. 4B) occupancy across the telomeric boundary region, suggesting a connection between KDACs and protein arginine methyltransferases in further regulating chromatin states. To gain further insights into the interplay between KDACs and Hmt1 at the telomeric boundary regions, we examined the effects of both Rpd3 and Sir2 on Hmt1-catalyzed H4R3 dimethylation in this region. We used a ChIP assay to determine the level of H4R3 dimethylation across this region, in both Δ*rpd3* (Fig. 6A) or Δ*sir2* cells (Fig. 6B). Our data indicate that the levels of dimethylated H4R3 across the telomeric boundary region is generally decreased in mutants that lack either Sir2 or Rpd3 (Figs. 6A and 6B, respectively), albeit to different extents: Δ*sir2* had more pronounced effects on the decrease in dimethylated H4R3 levels than Δ*rpd3* did. In sum, the absence of either Sir2 or Rpd3 influences the levels of H4R3 dimethylation at the telomeric boundary region.

## Discussion

Previously, we had discovered a role for Hmt1 in establishing and maintaining silent chromatin in the budding yeast [35]. Here, we have used a comprehensive reverse genetic screen to gain further insight into how Hmt1 influences these biological processes. Our genetic screen yielded genes that encode components of the Rpd3L complex. Rpd3, the most prominent member of this complex, has been demonstrated to play a role in establishing and maintaining the silenced state of chromatin at the telomere [14]. Using a ChIP assay, we demonstrated that Rpd3 recruitment across the telomeric boundary region is increased in Hmt1 loss-of-function mutants. Previously, we observed a loss of silencing in Hmt1 loss-of-function mutants. However, a double *rpd3* and *hmt1*-null mutant had increased telomeric silencing compared to the *hmt1*-null only mutant, indicating a dominant phenotype of the *rpd3*-null mutation. At the molecular level, more Sir2 is recruited to the telomeric boundary region in the double versus *hmt1*-null mutants. The Rpd3 ChIP data implied a potential change in the level of Rpd3 substrates across the telomeric boundary region. To this end, we tested for the effects of Hmt1 on a well-known Rpd3 substrate H4K5 [45] and correlated this change in Rpd3 occupancy in Hmt1 loss-of-function mutants with a decrease in the H4K5 acetylation across the telomeric boundary region from 0.6 to 2.8 Kb. We also tested for H4K16 acetylation in the *hmt1* mutants, given our previous and current observation of Sir2 occupancy change across the telomeric boundary region in these mutants. We found that H4K16 acetylation levels increased at the telomeric boundary regions in these mutants. To determine the degree of synergy between Hmt1 and the KDACs Rpd3 and Sir2 in establishing and maintaining telomeric silent chromatin, we examined the level of H4R3 dimethylation at the boundary regions in both *rpd3*-null and *sir2*-null mutants, and found that each KDAC is required for proper levels of dimethylated H4R3 levels at these regions, albeit to a different degree.

Large-scale reverse genetic screens have provided information useful in deciphering how specific genes contribute to a biological process [48]. A number of SGA screens have previously been performed; the most recent one examined approximately 5.4 million gene-pairs for genetic interactions [38]. While approximately 170,000 genetic interactions were identified in this recent study [38], only 55 had been found for Δ*hmt1.* A recent report by the Hartman group demonstrated that screens carried out using the conventional SGA query strain have the potential to produce many false negatives [36]. It can be inferred that many more genetic interactions for Δ*hmt1* could be identified using a query strain that reduces false negatives. Thus, we carried out an SGA screen using a query strain that was a re-engineered form of the conventional SGA query strain [36]; specifically, the newly engineered query strain is more effective in preventing mating-type-regulated auxotrophy escape and mating-type switching [36]. Using this newly engineered query strain increased the total number of genetic interactors identified for Δ*hmt1* by 2.5-fold, which is a change of magnitude well within the parameters described for this strain [36]. As expected, there was an overlap in the genetic interactions identified in our screen and the previous one, and our screen revealed a substantial number of new interactors. These include many additional genes within a pathway or complex that have been discovered using the conventional SGA query strain. For example, our screen identified five genes encoding components of the yeast Spt-Ada-Gcn-Acetyltransferase (SAGA) complex, whereas the screen using the conventional query strain found two genes that functions in that complex [38]. It is possible that one other reason for the differences in the quantity and identity of hits between our screen and the Boone lab screen, besides the use of a different query strain, is differing methodologies for scoring growth phenotypes. However, our SGA analysis provides an improvement in the genetic interaction profile of *HMT1*. With this information, we were able to more comprehensively map *HMT1* interaction networks, as well as to uncover new roles of *HMT1* in various biological processes, such as those in mitochondrial function, protein folding, and proteasome assembly and function. By assessing the level of physical connectivity between gene products based on the genes identified, we have been able to infer the potential new functions for *HMT1* in different pathways. This is because synthetic interactions among nonessential genes generally do not overlap with physical interactions between corresponding gene products [49].

The information from our SGA screen forms an important initial blueprint for dissecting the potential functional roles of Hmt1 in these biological pathways. As a proof of this principle, we decided to investigate the potential role of Hmt1 in Rpd3L complex function given that our gene ontology analysis approach had identified “Rpd3L complex” as enriched based on the total genetic interactions we identified from our SGA screen. While the majority of our Rpd3L interactions display a change in the growth phenotype, only *SAP30* and *PHO23* showed complete lethality with *HMT1*. It is unlikely that the synthetic lethality seen between *HMT1* and genes encoding Rpd3L complex components is due to the loss of silencing, since null mutations of SIR components are not lethal [50]. Rather, it may be attributed to the loss of regulation of Rpd3 activity or its targeting, thereby altering gene expression within a cell.

**Table 1:** Yeast strains used in this study.

Strain	Genotype	Reference
MYY192	*MATα, ura3–53, leu2–3,112, ade2–1, 01, can1–100, his3-*Δ*200, ADE2-TEL V-R, RDN1(18S)::URA3*	[42]
MYY210	*MATα, ura3–53, leu2–3,112, ade2–1, 01, can1–100, his3-*Δ*200, ADE2-TEL V-R, RDN1(18S)::URA3,* Δ*hmt1::KANMX4*	[35]
BY4741	*MATa, his3Δ1, leu2Δ0, met15Δ0, ura3Δ0*	[59]
MYY364	*MATα, ura3–53, leu2–3,112, ade2–1, 01, can1–100, his3-*Δ*200, ADE2-TEL V-R, RDN1(18S)::URA3,* Δ*hmt1::KANMX4* *hmt1(G68R)::LEU2*	[35]
MYY432	*MATa, his3Δ1, leu2Δ0, met15Δ0, ura3Δ0,* Δ*hmt1::KANMX6*	This Study
MYY648	*MATa, his3Δ1, leu2Δ0, met15Δ0, ura3Δ0,* Δ*hmt1::KANMX6, hmt1(G68R)::LEU2*	This Study
MYY653	*MATa, his3Δ1, leu2Δ0, ura3Δ0, can1Δ0, PGAL1-TADH1-PMFA1-his5+, lypΔ0, hmrΔ0::URA3ca,* Δ*hmt1::NATR*	This Study
MYY937	*MATa, his3*Δ*1 leu2*Δ*0 met15*Δ*0 ura3*Δ*0* Δ*sir2::KANMX4*	This Study
MYY941	*MATa, his3*Δ*1 leu2*Δ*0 met15*Δ*0 ura3*Δ*0,* Δ*hmt1::HIS3*	This Study
MYY979	*MATa, his3Δ1, leu2Δ0, met15Δ0, ura3Δ0, RPD3–13myc::KANMX6*	This Study
MYY987	*MATa, his3Δ1, leu2Δ0, met15Δ0, ura3Δ0, RPD3–13myc::KANMX6,* Δ*hmt1::HIS3*	This Study
MYY990	*MATa, his3Δ1, leu2Δ0, met15Δ0, ura3Δ0,* Δ*hmt1::HIS3, hmt1(G68R)::LEU2, RPD3–13myc::KANMX6*	This Study
MYY997	*MATa, his3*Δ*1 leu2*Δ*0 met15*Δ*0 ura3*Δ*0* Δ*rpd3::KANMX4*	This Study
MYY1007	*MATa, his3*Δ*1 leu2*Δ*0 met15*Δ*0 ura3*Δ*0* Δ*rpd3::KANMX4,* Δ*hmt1::HIS3*	This Study
MYY1285	*MATα, his3Δ1 leu2Δ0 ura3Δ0 can1Δ0::PGAL1-TADH1-PMFA1-his5+ lypΔ0 hmrΔ0::URA3ca* Δ*met15::NATR*	This Study
MYY1434	*MATα, ura3–53, leu2–3,112, ade2–1, 01, can1–100, his3-*Δ*200, ADE2-TEL V-R, RDN1(18S)::URA3,* Δ*hmt1::KANMX4,* *hmt1(G68R)::LEU2,* Δ*rpd3::HIS3*	This Study
MYY1402	*MATα, ura3–53, leu2–3,112, ade2–1, 01, can1–100, his3-*Δ*200, ADE2-TEL V-R, RDN1(18S)::URA3,* Δ*rpd3::KANMX4*	This Study
MYY1443	*MATα, ura3–53, leu2–3,112, ade2–1, 01, can1–100, his3-*Δ*200, ADE2-TEL V-R, RDN1(18S)::URA3,* Δ*hmt1::KANMX4,* Δ*rpd3::HIS3*	This Study

Interestingly, it has previously been shown that spreading of the SIR complex at telomeric boundary regions is antagonized by the presence of Rpd3 [14]. Moreover, Sir2 recruitment to the telomeric boundary region has been shown to be reduced in mutants that lack Hmt1 [35]. Given the newly found genetic connection between Δ*hmt1* and genes encoding the Rpd3L complex, we hypothesized that Hmt1 may influence recruitment of Rpd3, the catalytic subunit for the Rpd3L complex, to the telomeric boundary regions. This hypothesis is supported by our Rpd3 ChIP data, in which Rpd3 occupancy is increased in both null and catalytically inactive mutants of Hmt1. Moreover, these data indicate that the catalytic activity of Hmt1 restricts the recruitment of Rpd3 to these regions. Data from our telomeric silencing assay indicated that the Δ*rpd3* mutation is dominant over Δ*hmt1*, as mutants carrying dual Δ*rpd3*/Δ*hmt1* mutations display a similar silencing phenotype as that of Δ*rpd3* (more silenced than the wild-type), not that of Δ*hmt1* (less silenced than the wild-type). This is also supported by the change in Sir2 occupancy level in these Δ*rpd3*/Δ*hmt1* double mutants, in which Sir2 recruitment at the telomeric region displays a trend more similar to that of Δ*rpd3* than Δ*hmt1*. We note that these results do not distinguish whether such an increase in Rpd3 recruitment is simply a consequence of decreased Sir2 occupancy in the Hmt1 loss-of-function mutants, or the decrease in Sir2 occupancy seen in these mutants are due to an increase in Rpd3 recruitment. While we have not tested the role of Hmt1 in controlling the boundaries of the silenced domains at *HMR* and *HML* silent mating type loci, our previous work has demonstrated that Sir2 recruitment in Hmt1 loss-of-function mutants is decreased at the E silencer and immediately upstream of the α2 gene in *HML* and I silencer of *HMR* [35]. In a mutant that overexpresses Hmt1, increased Sir2 recruitment is observed only on the two regions of the *HML* locus [35]. While this evidence points to a likely role of Hmt1 in controlling the boundaries of the silenced domains at *HM* silent mating loci, more experiments must be done in order to elucidate the precise molecular mechanisms underlying this phenomenon at the silent mating loci.

**Table 2:** Oligonucleotides used for chromatin immunoprecipitation assay in this study.

Name	Description	Sequence	Reference
MCY1343	Tel 0.35 Forward	CTTTCTGGAATAGCGTTCGG	This Study
MCY1344	Tel 0.35 Reverse	ATCATAAACATAAGCGTATCCA	This Study
MCY273	Tel 0.6 Forward	CAGGCAGTCCTTTCTATTTC	[60]
MCY274	Tel 0.6 Reverse	GCTTGTTAACTCTCCGACAG	[60]
MCY275	Tel 1.4 Forward	AATGTCTTATCAAGACCGAC	[60]
MCY276	Tel 1.4 Reverse	TACAGTCCAGAAATCGCTCC	[60]
MCY277	Tel 2.8 Forward	CTGATCTGATGTTCTCACGC	[60]
MCY278	Tel 2.8 Reverse	TCTGTATGAGTCATCGAAGC	[60]
MCY61	ACT1 Forward	CGGTTCTGGTATGTGTAAAGCCG	This Study
MCY286	ACT1 Reverse	CATGATACCTTGGTGTCTTG	This Study
MCY384	No ORF Forward	GAAAAAGTGGGATTCTGCCTGTGG	[61]
MCY385	No ORF Reverse	GTTTGCCACAGCGACAGAAGTATAACC	[61]
MCY907	Tel 5.0 Forward	GGCTAGAAAAGCTTCAACATGGCCTTAC	[62]
MCY908	Tel 5.0 Reverse	CTCCAGCCTGCCTAAGACAAGCTATAG	[62]
MCY305	GAL1 Forward	TGCTAGATCGCCTGGTAGAG	[60]
MCY306	GAL1 Reverse	GCAAACCTTTCCGGTGCAAG	[60]

Based on our Rpd3 ChIP data, it is likely that an increase in Rpd3 recruitment to these regions would impact the acetylation state of histones, since Rpd3 substrates include a wide range of acetylation sites within histones H4 and H3 [45]. In mammalian cells, PRMT1 depletion decreases the abundance of acetyl-H4K5 in the upstream element of a developmentally regulated, erythroid-specific gene [47]. Indeed, we observed a decrease in the levels of acetyl-H4K5 between 0.6 Kb to 2.8 Kb of the telomeric repeats in *hmt1* loss-of-function mutants. This line of evidence supports our observation of increased Rpd3 recruitment at these regions in these mutants, as Rpd3 deacetylates acetyl-H4K5 [9]. Additional supporting evidence comes from our ChIP analysis of acetyl-H4K16, which revealed an increase in the levels of acetyl-H4K16 in this region.

Previous studies in mammalian systems have established that KATs and PRMTs have synergistic activities [51], [52]. However, synergy between KDACs and PRMTs remains to be shown. Our data revealing that mutants lacking either Rpd3 or Sir2 display different degrees of dimethylated H4R3 at the telomeric boundary region are suggestive of cooperative interactions between HATs, KDACs, and PRMT in promoting proper chromatin states. While both Rpd3 and Sir2 mutants exhibited a decrease in H4R3 dimethylation at the telomeric boundary regions, the reduction in Sir2 mutants was higher than that in the Rpd3 mutants. A potential explanation for this relates to the difference in the levels of specific acetylated histone H4 residues in these mutants. A detailed *in vitro* study of the four acetylatable lysine residues in the N-terminal tail of histone H4 reveals that each acetylated histone H4 has a different ability to act as a substrate for Hmt1/PRMT1 [53]. This study demonstrated that acetyl-H4K16 is the least effective substrate for Hmt1 binding, followed by acetyl-H4K5, acetyl-H4K12, and acetyl-H4K8 (i.e. the most effective substrate) [53]. As such, the decrease in dimethyl-H4R3 in mutants lacking Rpd3 may be attributable to an overall higher level of acetyl-H4K5 in these cells, making these histones slightly more attractive for Hmt1; in mutants that lack Sir2, the levels of acetyl-H4K16 would likely be higher; and these are the least effective substrate for Hmt1 binding.

In conclusion, our data indicate that loss of either Hmt1 or its catalytic activity influences Rpd3 occupancy at the telomeric boundary region, and, as a consequence, the levels of H4K5 acetylation in this region. Given the proposed mechanism for how Rpd3 antagonizes Sir2 action at these regions [16], it is possible that our previous observation of a decrease in Sir2 recruitment at these regions in the Hmt1 loss-of-function mutants was due to a change in Rpd3 occupancy in these same mutants. While the catalytic activity of Hmt1 is known to be necessary for regulating the recruitment of Rpd3 to the telomeric boundary regions, it is unclear how this occurs. One possibility is that Hmt1 is required to methylate H4R3 at such regions, and that the resulting methylated H4R3 then triggers a cascade of other histone modification events. Indeed, H4R3 methylation by mammalian PRMT1 has been shown to be important for subsequent histone modifications [47]. Given the high degree of conservation between mammalian PRMT1 and yeast Hmt1, it is likely that H4R3 methylation by Hmt1 accomplishes the same feat. An alternative possibility is that Hmt1 may methylate one of the regulatory proteins responsible for recruiting Rpd3 to the telomeric boundary regions. Given that methyl marks catalyzed by Hmt1 are the necessary “off” switch for protein-protein interactions [22], [23], it is possible that in the absence of Hmt1, or its catalytic activity, Rpd3 is not able to properly disengage itself from the protein that recruits Rpd3 to the telomeric boundary region. It should be noted that the regulatory protein may be histone H4 itself, or another modified form of histone H4. Further studies are needed to distinguish these possibilities.

## Materials and Methods

### Yeast Strains Used in this Study

All yeast strains used in this study are listed in Table 1. Cells were grown at 30°C on YPD medium (1% yeast extract, 2% bactopeptone, 2% glucose, w/v) unless otherwise stated.

### Synthetic Genetic Array (SGA)

The SGA methodology was performed essentially as described previously [54], with the following modifications: 1) a strain background (15578-1.2b, kind gift from John Hartman IV) different from the original SGA query strain background was used to construct the Δ*hmt1* query strain. 2) images of each plate were taken with a CCD camera (Bio-Rad) to assess the degree of cell growth for each double mutant. These images were then processed using customized MATLAB code adopted from studies done by Collins et al. Three independent screens were carried out to identify all of the genes that interact Δ*hmt1*. These interactions were then scored based on the percentage difference in the colony size when compared to a control query strain containing only deletion library mutation. Based on the results from all three screens, a *p-*value was assigned to each genetic interaction tested. The following criteria were used to score and filter the raw data for inclusion in the final dataset: 1) the double mutant had a corresponding control mating present on the final selection medium; and 2) any interactions with dubious or putative ORFs were removed; and 3) the double mutant exhibited a growth difference of 50% or more relative to the control, and the *p*-value was < or  = 0.001. The genetic interaction network was created using Cytoscape [55], using only the genes identified in this study. A number of SGA hits from this study were verified by the tetrad analysis (Fig. S1); this includes ones that were identified from the Boone lab study only, as well as the ones that were identified in our study but not from the Boone lab ones, or the ones that were identified in both studies.

### Chromatin Immunoprecipitation (ChIP)

ChIP procedures were performed as described previously [35] with three biological samples (n = 3). For each biological replicate, qPCR was performed in triplicates. For qPCR, 2 µl of DNA sample (input or IP) were used in 20 µl reactions with 500 nM final concentration of each primer and Bio-Rad iQ SYBR Green Supermix or Invitrogen Power SYBR Green PCR Master Mix. Table 2 shows the primers used in quantitative PCR experiments. For each immunoprecipitation of myc-epitope tagged protein, 10 µl of monoclonal a-myc (9E11, Thermo-Fisher) was pre-coupled to 40 µl of Protein-A sepharose beads. In the case of modified histones, either 4 µl of α-acetyl-H4K5 (Millipore cat#07-327), 5 µl α-acetyl H4K16 (Millipore cat #07-329), 5 µl of α-dimethyl-H4R3 (Active Motif cat#39706), or 1 µl of α-Sir2 (kind gift from Danesh Moazed) was pre-coupled to 40 µl of Protein A sepharose beads for each immunoprecipitation.

### Silencing Assay

Null mutations of *HMT1* or *RPD3* were constructed in a published yeast strain with *ADE2* integrated at the right telomere of chromosome 5 [42]. The strains were grown overnight in YEPD and the cells were collected and washed once with sterile water, followed by resuspension in 1× volume of sterile water. Cell density was normalized and 10-fold serial dilutions of each strain were spotted (5 µl) onto synthetic dextrose media with a final adenine concentration of 10 mg/L. Plates were incubated at 30°C for 2 days and then moved to 4°C for 3–4 days to allow the development of red pigment on colonies.

## Supporting Information

Figure S1 Tetrad analysis was used to confirm *HMT1* interactors from the SGA analysis: A) *HMT1* interactors found in the Boone lab study only; B) *HMT1* interactors identified in both the Boone lab study and in this study; C) *HMT1* interactors found in this study that had negative synthetic interactions; and D) *HMT1* interactors found in this study that had positive synthetic interactions. Colonies represents non-query single mutant is marked by a white square and the double mutant (both query and non-query) is marked by a white circle.
(TIF)

Table S1 The list of all genes that display a genetic interaction with *HMT1* as described by the current study. The growth difference ranges from +1 (increasing colony size) to -1 (decreasing colony size) and the *p*-value for each interaction is also shown. Only genes that passed the *p-*value criteria are included in this table.
(PDF)

Table S2 The unfiltered list of SGA data from three independent screens. Control Average: average colony size for control query mutants; Control S.D.: standard deviation of control query colonies, Query Average: average colony size for Δ*hmt1* query mutants. Query S.D.: standard deviation of Δ*hmt1* query colonies. No. Sets: number of replicates scored. Visual Significance: binary assessment of replicate quality; 1 = good, 0 = poor.
(XLSX)
