# Correction: Recruitment of Rpd3 to the Telomere Depends on the Protein Arginine Methyltransferase Hmt1

**DOI:** 10.1371/journal.pone.0120968

**Published:** 2015-03-20

**Authors:** The PLOS ONE Staff

The following information is missing from the Funding statement: This work was also supported by an NSF award (MCB-1051350) to MCY.

The complete, correct Funding statement is as follows: This work was supported by a Scientist Development grant (0830279N) from the American Heart Association and an NSF award (MCB-1051350) to MCY. The publication costs of this article were supported by the Julian Park Fund, College of Arts and Sciences, University at Buffalo. The funders had no role in study design, data collection, data interpretation, or the decision to prepare and publish of the manuscript.
