# Supplementary figures and images for: Recruitment of Rpd3 to the Telomere Depends on the Protein Arginine Methyltransferase Hmt1
Source: PLoS One. 2012 Aug 31;7(8):e44656. doi: 10.1371/journal.pone.0044656 (PMC3432115; doi:10.1371/journal.pone.0044656)

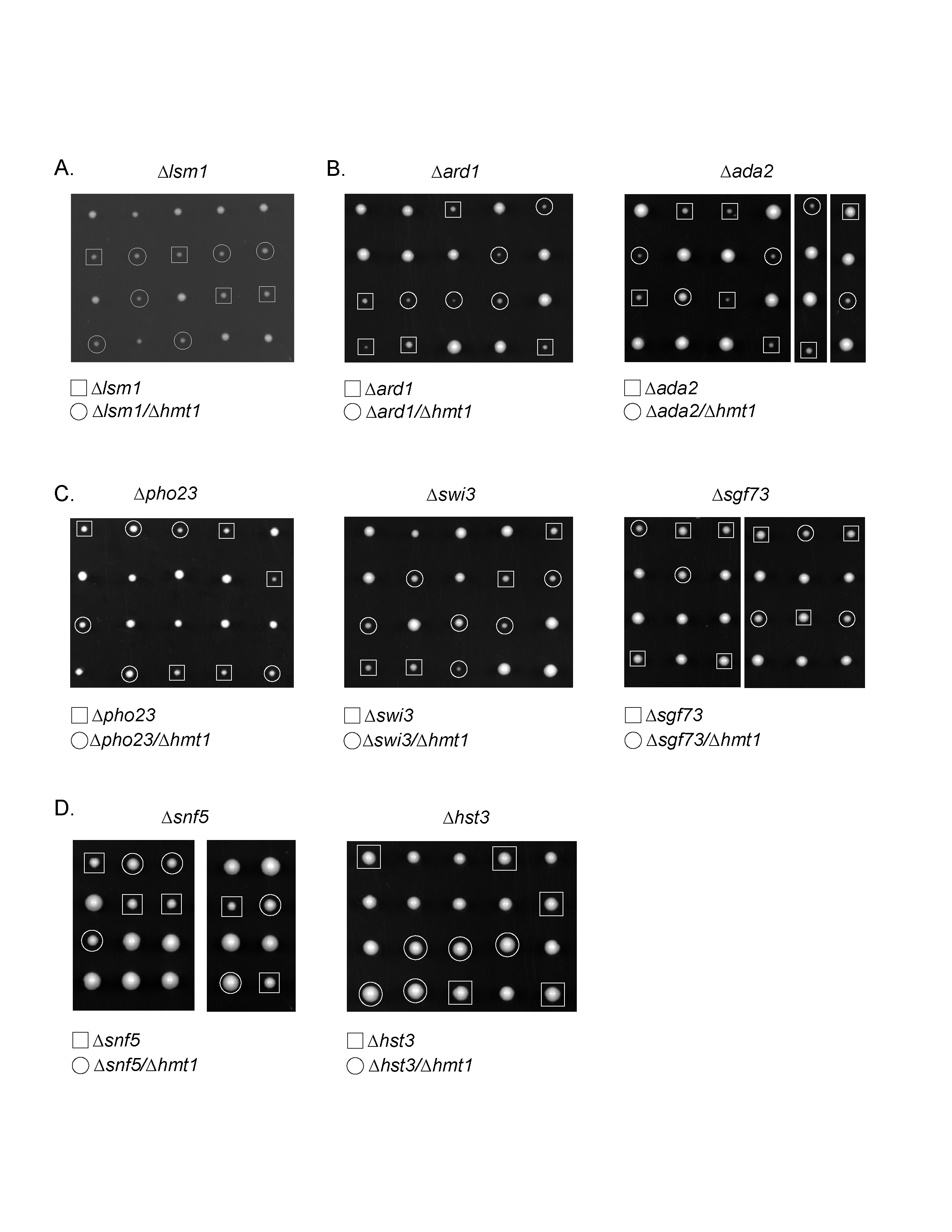

Supplement: Figure S1 — Tetrad analysis was used to confirm HMT1 interactors from the SGA analysis: A) HMT1 interactors found in the Boone lab study only; B) HMT1 interactors identified in both the Boone lab study and in this study; C) HMT1 interactors found in this study that had negative synthetic interactions; and D) HMT1 interactors found in this study that had positive synthetic interactions. Colonies represents non-query single mutant is marked by a white square and the double mutant (both query and non-query) is marked by a white circle. (TIF) [file pone.0044656.s001.tif]
