# Supplementary material for: Recruitment of Rpd3 to the Telomere Depends on the Protein Arginine Methyltransferase Hmt1
Source: PLoS One. 2012 Aug 31;7(8):e44656. doi: 10.1371/journal.pone.0044656 (PMC3432115; doi:10.1371/journal.pone.0044656)
Supplement: Table S1 — The list of all genes that display a genetic interaction with HMT1 as described by the current study. The growth difference ranges from +1 (increasing colony size) to -1 (decreasing colony size) and the p-value for each interaction is also shown. Only genes that passed the p-value criteria are included in this table. (PDF) [file pone.0044656.s002.pdf]

Y-name	Alias	Growth Difference	p-value
YDR448W	*ADA2*	-1	0.00417
YGR204W	*ADE3*	-1	0.00906
YER170W	*ADK2*	-0.9424	0.00321
YNL270C	*ALP1*	-1	0.00018
YBR288C	*APM3*	0.5185	0.00336
YHR013C	*ARD1*	-1	6.00E-05
YER069W	*ARG5,6*	-1	0.00194
YML099C	*ARG81*	0.7013	0.00025
YGL157W	*ARI1*	-0.6604	0.0026
YGL148W	*ARO2*	-0.9948	0.00041
YBR200W	*BEM1*	-1	1.00E-05
YER155C	*BEM2*	-1	0
YER155C	*BEM2*	-1	0.11656
YNL271C	*BNI1*	-1	0.00089
YGR134W	*CAF130*	0.6711	0.00862
YPL178W	*CBC2*	-0.9363	0.00031
YBR131W	*CCZ1*	1.3307	0.00186
YPL132W	*COX11*	0.6004	0.00464
YMR078C	*CTF18*	1.2622	0.00271
YPL181W	*CTI6*	-0.9469	9.00E-05
YEL027W	*CUP5*	-1	0.03757
YAL013W	*DEP1*	-1	0.05717
YDR069C	*DOA4*	-1	0.01032
YDR424C	*DYN2*	0.7667	0.00959
YHR021W-A	*ECM12*	0.7133	4.00E-05
YBR033W	*EDS1*	-1	0.1096
YHR193C	*EGD2*	0.7265	0.00582
YER044C	*ERG28*	-1	0.00395
YLR056W	*ERG3*	-0.9215	0.00118
YOR033C	*EXO1*	-1	2.00E-05
YIL065C	*FIS1*	0.5582	0.00073
YML074C	*FPR3*	0.5228	0.00655
YGR252W	*GCN5*	-1	0
YNL153C	*GIM3*	-0.6891	0.00061
YML094W	*GIM5*	-0.9085	0.00061
YJR090C	*GRR1*	-1	0.41582
YBR121C	*GRS1*	-0.9824	0.0019
YPL254W	*HFI1*	-1	4.00E-05
YML075C	*HMG1*	2.9879	0.00678
YOR025W	*HST3*	0.5472	5.00E-05
YER078C	*ICP55*	0.6798	0.00424
YJL073W	*JEM1*	0.5306	0.00866

YDR532C	*KRE28*	-1	0.00018
YCL005W	*LDB16*	-1	0.0186
YBL006C	*LDB7*	-1	0.00041
YIR034C	*LYS1*	-1	0.00099
YGR292W	*MAL12*	0.7422	0.00933
YJL102W	*MEF2*	0.5676	0.00368
YOR211C	*MGM1*	-0.7093	0.00442
YHR015W	*MIP6*	-0.9635	0.00253
YNL284C	*MRPL10*	-0.8216	0.00384
YKR006C	*MRPL13*	-0.8122	0.00516
YDR322W	*MRPL35*	-0.6822	3.00E-05
YPR100W	*MRPL51*	-0.7208	0.00962
YOL116W	*MSN1*	0.691	0.00701
YDR335W	*MSN5*	-0.9718	4.00E-05
YDR176W	*NGG1*	-1	0.1222
YML118W	*NGL3*	0.6789	0.00079
YPR072W	*NOT5*	0.6124	0.00128
YGL151W	*NUT1*	-0.9626	0.00039
YGL038C	*OCH1*	-1	0.09213
YOR036W	*PEP12*	-0.9351	0.00108
YGL153W	*PEX14*	-1	0.00028
YJL179W	*PFD1*	-1	0.37715
YNL097C	*PHO23*	-1	0.00204
YOL001W	*PHO80*	-0.9602	5.00E-05
YML107C	*PML39*	0.5763	0.00999
YPL144W	*POC4*	-1	0.01867
YNR052C	*POP2*	0.5609	0.00587
YPL188W	*POS5*	-1	0
YGR135W	*PRE9*	-0.8355	0.0012
YER095W	*RAD51*	0.5531	0.00492
YGL058W	*RAD6*	-0.9551	0.00488
YBL033C	*RIB1*	-1	0.00084
YHL027W	*RIM101*	-0.7184	5.00E-05
YMR154C	*RIM13*	-0.5818	0.00267
YOR275C	*RIM20*	-0.655	0.00527
YGL046W	*RIM8*	-0.5905	0.0065
YEL072W	*RMD6*	-0.999	2.00E-05
YBR084C-A	*RPL19A*	0.5464	0.00654
YDL075W	*RPL31A*	-1	0.0065
YGL147C	*RPL9A*	-0.9481	0.00355
YLR357W	*RSC2*	-1	0.10667
YBR095C	*RXT2*	-0.9965	8.00E-05
YDL076C	*RXT3*	0.6555	0.00388

YMR263W	*SAP30*	-1	0.00011
YIL076W	*SEC28*	1.344	0.00578
YGL066W	*SGF73*	-0.9159	8.00E-05
YOL004W	*SIN3*	-1	2.00E-05
YDL047W	*SIT4*	-0.6395	0.00307
YCR024C	*SLM5*	-0.5313	0.00294
YOR290C	*SNF2*	-0.9678	3.00E-05
YHL025W	*SNF6*	-1	0.01683
YLR025W	*SNF7*	-1	0.04328
YMR096W	*SNZ1*	0.6809	0.00936
YGL127C	*SOH1*	1.0458	0.00129
YHR014W	*SPO13*	-1	6.00E-05
YER161C	*SPT2*	-0.9726	0.00305
YNL025C	*SSN8*	3.7261	0.00968
YCL032W	*STE50*	-0.9974	0.00011
YLR372W	*SUR4*	-0.9855	0.00026
YBR231C	*SWC5*	-1	0.02687
YJL176C	*SWI3*	-0.9802	0.00203
YDR079C-A	*TFB5*	-0.5832	0.0022
YDL185W	*TFP1*	-1	0.02725
YHR025W	*THR1*	-0.7992	0.00644
YAL016W	*TPD3*	-1	0.11161
YDR007W	*TRP1*	18.1934	0.00443
YMR071C	*TVP18*	0.6407	0.00573
YBR058C	*UBP14*	7.2239	0.00458
YBR173C	*UMP1*	-1	0.00028
YKL080W	*VMA5*	-1	1.00E-05
YGR020C	*VMA7*	-1	0.02928
YEL051W	*VMA8*	-1	0.04396
YKL119C	*VPH2*	-0.8471	0.00165
YPL045W	*VPS16*	-0.9268	0.00659
YMR077C	*VPS20*	-0.9566	0.00821
YOR083W	*WHI5*	0.5228	5.00E-05
YPL239W	*YAR1*	0.5351	0.00115
YDR056C	*YDR056C*	-1	0.00016
YOL128C	*YGK3*	0.5663	0.00175
YGL159W	*YGL159W*	-0.8336	0.00219
YHR029C	*YHI9*	-1	2.00E-05
YGL161C	*YIP5*	-1	0.00252
YJR011C	*YJR011C*	-0.991	0
YLR200W	*YKE2*	-1	0.00144
YPR063C	*YPR063C*	0.538	0.00052
YPR071W	*YPR071W*	0.6462	0.00682

YPR084W	*YPR084W*	0.5744	0.00185
YGL164C	*YRB30*	-0.9629	0.0018
